# Probing Individual Particles in Aquatic Suspensions by Simultaneously Measuring Polarized Light Scattering and Fluorescence

**DOI:** 10.3390/bios11110416

**Published:** 2021-10-25

**Authors:** Zhihang Xiong, Hongjian Wang, Jiajin Li, Ran Liao, Haoji Mai, Caizhong Guan, Zhiming Guo, Shangpan Yang, Yan Chen, Biwang Liu, Tong Liu, Hongyi Li, Wenzheng Ding, Yaguang Zeng, Hui Ma

**Affiliations:** 1 Institute for Ocean Engineering, Shenzhen International Graduate School, Tsinghua University, Shenzhen 518055, China; 2112055011@stu.fosu.edu.cn (Z.X.); whj20@mails.tsinghua.edu.cn (H.W.); lijiajin21@mails.tsinghua.edu.cn (J.L.); gzm20@mails.tsinghua.edu.cn (Z.G.); 201971160@yangtzeu.edu.cn (Y.C.); lt20@mails.tsinghua.edu.cn (T.L.); 2 Department of Photoelectric Technology, Foshan University, Foshan 528000, China; 20180300309@stu.fosu.edu.cn (H.M.); 2111955010@stu.fosu.edu.cn (C.G.); 20190300114@stu.fosu.edu.cn (S.Y.); 20170240406@fosu.edu.cn (B.L.); 20190220407@stu.fosu.edu.cn (H.L.); dingwzh@m.scnu.edu.cn (W.D.); Zeng_YG@fosu.edu.cn (Y.Z.); 3 Department of Biomedical Engineering, Tsinghua University, Beijing 100084, China; 4 Guangdong Research Center of Polarization Imaging and Measurement Engineering Technology, Shenzhen International Graduate School, Tsinghua University, Shenzhen 518055, China; mahui@tsinghua.edu.cn; 5 Department of Physics, Tsinghua University, Beijing 100084, China

**Keywords:** polarized light scattering, fluorescence, suspended particles

## Abstract

Suspended particles play a significant role in aquatic systems. However, existing methods to probe suspended particles have several limitations. In this paper, we present a portable prototype to in situ probe individual particles in aquatic suspensions by simultaneously measuring polarized light scattering and fluorescence, aiming to obtain an effective classification of microplastics and microalgae. Results show that the obtained classification accuracy is significantly higher than that for either of these two methods. The setup also successfully measures submicron particles and discriminates two species of *Synechococcus*. Our study demonstrates the feasibility of simultaneously measuring polarized light scattering and fluorescence, and the promising capability of our method for further aquatic environmental monitoring.

## 1. Introduction

Suspended particles, both biological and non-biological, play a significant role in natural aquatic systems [1]. These particles are generally observed through optical microscopy [2]. However, measurements based on microscopy are time-consuming and difficult to utilize in situ, which limits their further application in field testing [3]. The light scattering technique has been used to obtain the physical information of particles, such as the overall features of size, shape, and refractive index [4]. However, light scattering is traditionally measured through bulk volume scattering, which limits its usefulness for classification [5].

Polarized light scattering has been reported to be sensitive to the microstructure of particles and has the potential to differentiate different categories of microparticles [6]. Furthermore, polarized light detection has shown potential in the measurement of harmful microalgae [7], cancerous tissues [8], and atmospheric aerosols [9]. Polarized light backscattering at 120° has been confirmed in a previous report [10] to characterize the microstructures of different particles, while not sensitive to the size of microparticles. However, bottlenecks are often met during experiments because of a lack of ability to identify biological and non-biological particles.

The intracellular pigment, such as chlorophyll, can be measured and characterized using fluorescence spectrum analysis [11], and the fluorescence emission can differentiate some categories of algae [12]. Flow cytometry measures individual cells based on hydrodynamic focusing and records the intensity of light scattering and fluorescence [13,14]. However, the complicated pre-treatment and flow blockage limit its application [15], and some form of fine classification method is always needed in the aquatic research community.

**Figure 1:**
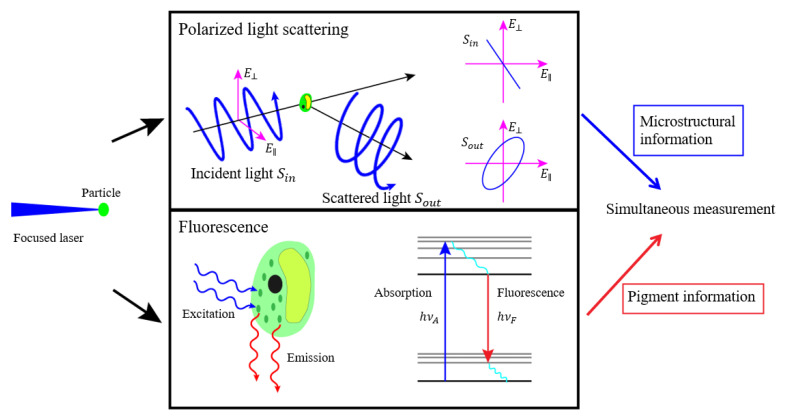
The schematic diagram of the physical mechanism.

In this paper, we present a new method to probe individual particles through light focusing, and measure the polarized light scattering and fluorescence of the individual particles. The physical mechanism is presented in Figure 1, which illustrates the processes that occur when a laser beam is focused on an aquatic suspension. Individual particles randomly pass through the focus area. Polarized light scattering and fluorescence occur as a result of the interaction between the individual particles and the incident light. When incident polarized light $S_{in}$ irradiates a single particle, the amplitude and phase of the electric field vector will both change in the directions of the electric field, denoted as $E_{⊥}$ and $E_{\left|\right|}$*,* resulting in the change of the polarization state of scattered light $S_{out}$. The polarization state of the scattered light provides microstructural information about the particles. Pigments in algae, such as chlorophyll, absorb the energy of the incident photons $hv_{A}$ and emit photons with energy $hv_{F}$. The wavelength and intensity of the emitted fluorescence indicates the composition and content of the intracellular pigment.

Therefore, the polarized light scattering and fluorescence of the individual particles provide the microstructural and pigment information, which aid in the classification of different particles. In this work, a portable prototype is built based on this theory, and several experiments are conducted to test the feasibility of the prototype using some biological and non-biological samples. We also examine the ability of the prototype to classify particles with submicron size, including *Synechococcus*, whose size is about one micron. The results demonstrate that the prototype can adequately classify these different particles with the combination of polarized light scattering and fluorescence measurement.

## 2. Methods

### 2.1. Samples

Several kinds of microparticles were measured in this work, including biological and non-biological particles. As for the non-biological particles, they were as follows: a kind of smooth polystyrene microspheres with the diameter of 10 μm (PS10, Tianjin Big Goose Scientific Co. Ltd., Tianjin, China), a kind of smooth polystyrene microspheres with the diameter of 0.5 μm (PS0.5, Suzhou Nanomicro Technology Co. Ltd., Suzhou, China), a kind of rough polystyrene microspheres with the same diameter but 100-nm uniform pores on their surface (PS10-100, Suzhou Nanomicro Technology Co. Ltd.), a kind of smooth silicon dioxide microspheres with 10 μm diameter (SiO2, Tianjin Big Goose Scientific Co. Ltd.), and a kind of smooth 10-μm polystyrene microspheres containing fluorochrome with the exciting wavelength from 410 nm to 570 nm (PS10-F, Tianjin Big Goose Scientific Co., Ltd.). All these particles were suspended in water individually, and the concentrations of these microspheres in the concentrate were 250 mg/10 mL. During the experiments, 50 μL of the concentrate was sampled and diluted by 200 mL distilled water. Then the diluted sample was added into the sample pool and separately tested.

Four categories of microalgae from different phyla were compared during experiments. *Phaeodactylum tricornutum* (P.T., Bacillariophyta) is rich in fatty acids and is an important raw material for biodiesel [16]. P.T. is oval, with a length of 8 μm and a width of 3 μm. *Dunaliella salina* (D.S., Chlorophyta) produces rich carotenoids, and is widely distributed in ocean, lakes, and other saline environments [17]. D.S. is elliptical, with a length of 18~28 μm and a width of 9.5~14 μm. *Phaeocystis globosa* (P.G., Chrysophyta) and *Microcystis aeruginosa* (M.A., Cyanophyta) are two potentially harmful algae, and both are likely to cause toxic blooms [18,19]. Both two algae are spherical, and both are easily gathered during blooms; the diameter of a single cell of P.G. is 3~5 μm and that of M.A. is 3~7 μm.

Different species of *Synechococcus* were studied, including *Synechococcus* 805 (S-805) and WH7803 (S-WH7803); they are elongated with a size of 0.5~2 μm. They are one of the smallest and most important cyanobacteria in seawater, and related studies have been conducted for decades [20,21].

All of the microalgae were taken at their logarithmic growth state. S-WH7803 was provided by Shanghai Guangyu Biological Technology Co. Ltd. (Shanghai, China), and other microalgae were provided by Freshwater Algae Culture Collection at the Institute of Hydrobiology, Chinese Academy of Sciences.

### 2.2. Experimental Setup

**Figure 2:**
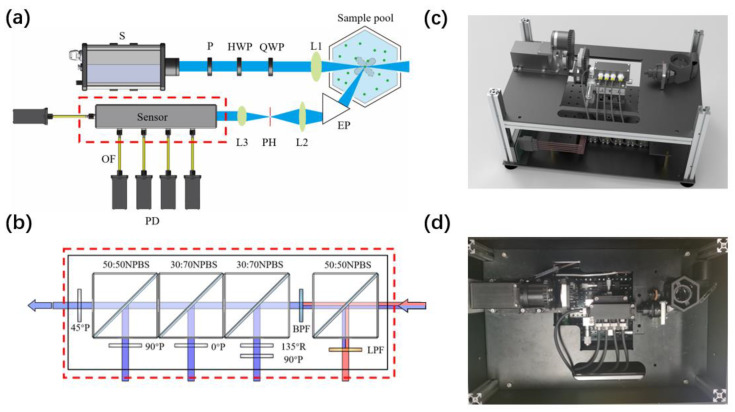
(**a**) Schematic of experimental setup. Laser source (S); linear polarizer (P); half-wave plate (HWP); quarter-wave plate (QWP); convex lenses (L1, L2, and L3); equilateral prism (EP); pinhole (PH); optics fiber (OF), photoelectric detector (PD). (**b**) Analyzer of the polarization and fluorescence. Non-polarizing beam splitter cube (NPBS); long-pass filter (LPF); bandpass filter (BPF); quarter-wave plate (R); polarizer (P); photoelectric detector (PD). (**c**) Three-dimensional model of the prototype. (**d**) Top view of the prototype.

The experimental setup is shown in Figure 2, where the light source is the laser diode S with a wavelength of 445 nm (300 mW, Δλ = 3 nm). The wavelength of 445 nm is close to the peaks of the absorption spectrum of chlorophyll-a (Chl-a) in vivo [22]. The linearly polarized light is obtained after the linear polarizer P, and the light can be modulated to different polarization states with a half-wave plate (HWP) and a quarter-wave plate (QWP). Note that the polarization state of the incident light can be selected depending on the situation, as different polarization states may provide different forms of physical information about the target particle. The polarization state of the incident light can be optimized to achieve the best classification through the modulation with the two wave plates [23]. In this work, we used a 45° linearly polarized light as the incident light, which works well in the cases examined here, although it is not the best.

Because the intrinsic fluorescence and scattered light of an individual particle are weak, the light focusing technique is applied to make the illumination more intense. The convex lens (L1) in front of the sample pool is used to control the illumination area and strengthen the energy density of the incident light. The diameter of the airy disk of the focused light is theoretically 10 μm, and the intersection volume between the incident and the receiving optical paths is the scattering volume.

During the experiment, the test sample is placed in the sample pool, with a black cover to block the background light. The stirrer rotates at the speed of 100 rounds per minute in order to keep the particles suspended in the water. After the interaction between the incident polarized light and suspended particles in the scattering volume, the polarized light scattering and fluorescence are emitted simultaneously. Then the light is received at the solid angles whose central angle is at 120 degrees of the backward scattering angle, and the received light accounts for 0.014 Steradian of the scattered light and fluorescence. Assuming that the emitted fluorescence is distributed evenly in all directions, the energy received accounts for 0.11% of the total fluorescence.

Then the scattered light at 120° is deflected through the equilateral prism (EP). The pinhole (PH) is adjusted to be coincident with the point where the light from the scattering volume is focused by the convex lenses (L2) in order to block the stray light outside the scattering volume. After that, the scattered light is collimated by the convex lenses (L3). 

The scattering volume can be confined to less than 0.01 microliters by reducing the focal spot size of the incident light and the size of the pinhole. When the concentration of particles in the water is lower than $10^{5}$ particles per millilitre, there is only one particle at most in the scattering volume. Therefore, for this proposed technique, if the concentration is not too high, the scattering and fluorescence properties of individual particles can be measured once the particles pass through the scattering volume.

The sensor was designed to analyze the polarization state of the scattered light and fluorescence based on the division-of-amplitude method [10,24], as shown in Figure 2b. The light is firstly split into two light beams using a 50:50 non-polarizing beam splitter cube (NPBS). A long-pass filter (LPF, passing range: >460 nm) is employed to block elastic scattering while allowing red-shifted fluorescence into the photoelectric detector (PD, spectral response range: 300–950 nm) with a radiant sensitivity of 5 V/nW. The remaining light passes a bandpass filter (BPF) with a central wavelength of 445 nm and 10 nm bandwidth to the polarization states analyzer (PSA).

The PSA consists of two 30:70 NPBSs and one 50:50 NPBS in order to split the beam into four parts. The left-handed circularly polarized component, horizontally polarized component, vertically polarized component, and 45° linearly polarized component are obtained through a 135° quarter-wave plate followed by a 90° polarizer, 0° polarizer, 90° polarizer, and 45° polarizer, respectively. Finally, the analyzed lights are converted by PDs to voltages and recorded by a data acquisition card as $I_{L},\;I_{0},\;I_{90},\;$and $I_{135}$, respectively. The practical prototype was built based on the principle above, and the structural layout is shown in Figure 2c,d. In this work, all the experiments were conducted using this built prototype. This prototype can be applied in laboratory settings, on ships, and in various other settings.

### 2.3. Data Analyzing Method

The Stokes vector, used to describe the polarization state of the light beam, is expressed as $S={\left[I,\;Q,\;U,\;V\right]}^{⊤}$, where *I* is the light intensity, and *Q*, *U*, and *V* are the residual intensities of the 0° linear polarization, 45° linear polarization, and right-handed circular polarization, respectively. The polarization components *q*, *u*, and *v* are defined by Equation (1) and range from −1 to 1. The degree of polarization (DOP), which is calculated using Equation (2), ranges from 0 to 1, and is used to evaluate the polarization proportion of the light.

(1) $$q=\frac{Q}{I},\;u=\frac{U}{I},\;v=\frac{V}{I},$$

(2) $$\mathrm{DOP}=\sqrt{q^{2}+u^{2}+v^{2}},$$

The voltage outputs of the four PDs are represented by the vector $I_{\mathrm{PSA}}={\left[I_{L},\;I_{0},I_{90},\;I_{135}\right]}^{⊤}$; the calibrated scattered Stokes vector$\;S_{s}$ is calculated from $I_{\mathrm{PSA}}$ and the instrumental matrix of analyzer $A_{PSA}$, as shown in Equation (3),

(3) $$S_{s}={\left(A_{PSA}\right)}^{-1}\times I_{PSA}.$$

Usually, the $A_{PSA}$ is obtained first by solving the equation above. For example, the Stokes vectors S_s, of the incident polarized light can be measured and known in advance by using Thorlabs PAX1000VIS(/M). We then record the $I_{\mathrm{PSA}}$ for each incident polarized light to form a series of equations according to Equation (3). By solving these equations, we obtained the instrumental matrix, $A_{PSA}$. After this calibration, the errors of the measured *q*, *u*, and *v* were estimated to be less than 3%.

The measured fluorescence light intensity in PD is denoted as $F_{m}$, which is the integral intensity from the wavelength 460 nm to 950 nm. Considering that the measured fluorescence intensity is related with the spatial location of particles in the scattering volume, then the calibrated fluorescence intensity, *F*, is defined as Equation (4) in order to remove this effect. Equation 4 presents the ratio of the measured fluorescence light intensity $F_{m}$ to the non-polarized scattered intensity *I*,

(4) $$\;F=\frac{F_{m}}{I}.$$

After the experiments, every sample can be characterized with the polarization parameters and fluorescence intensity. To better visualize and analyze our data, some machine learning algorithms were applied. Linear discrimination analysis (LDA) is a supervised dimensionality reduction algorithm [25]. The essential goal of LDA is to find an optimal transformation matrix to project the original high-dimension measured data to a low-dimension embedding subspace, by suppressing the difference within same class and enlarging the difference between classes. Different from LDA, the support vector machine (SVM) can be applied for classification task by projecting the raw data to a higher dimension [26], by use of the kernel method. Additionally, the hyper-parameter optimization is applied to improve the generalization ability of the obtained classifier. In this work, we used these two algorithms to classify the particles by using the simultaneously measured polarization parameters and fluorescence intensity.

## 3. Results

### 3.1. Non-Biological Particles

**Figure 3:**
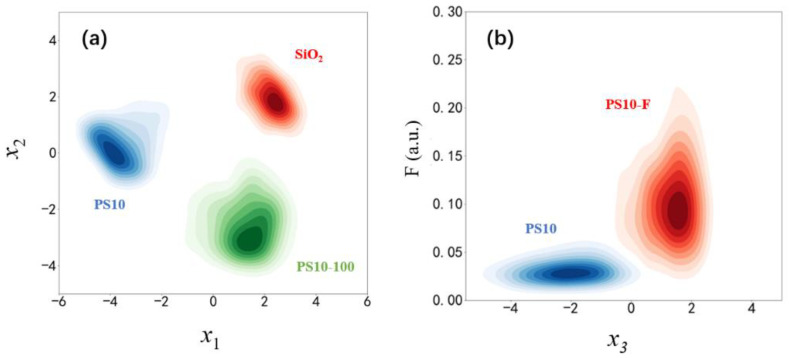
Distributions of four categories of non-biological particles. (**a**) Distributions of the smooth PS microspheres of 10 μm (PS10), the rough PS microspheres of 10 μm with 100-nanometer uniform holes on them (PS10-100), and the smooth silicon dioxide microspheres of 10 μm (SiO2). (**b**) Distributions of the smooth PS microspheres of 10 μm (PS10) and the smooth fluorescent PS microspheres of 10 μm with fluorochrome inside (PS10-F). *x_1_*, *x*_2_, and *x*_3_ are polarization parameters obtained by LDA, and F is the fluorescence-related value.

Four categories of non-biological particles were first illuminated by 45° linearly polarized light to test the feasibility of the experimental setup, that is, PS10, PS10-100, SiO_2_, and PS10-F. Firstly, the dimension of the measured five-dimensional polarization parameters, [*I*, *q*, *u*, *v*, *DOP*], was transformed into one dimensional projected parameter using the LDA algorithm. Every $x_{i}$ hereinafter is equal to the linear transformation of these five parameters, which can be represented as $x_{i}=f\left(I,\;q,\;u,\;v,\;\mathrm{DOP}\right)$. $x_{1}$ and $x_{2}$ are two new projected parameters that can help to achieve good classification among these three categories, as shown in Figure 3a. From Figure 3a, we can see that the polarization parameters can effectively differentiate particles with different microstructures and materials.

Since PS10 and PS10-F both have smooth surfaces and similar structures, the distributions of $x_{3}$ in Figure 3b have some overlap, but F values for PS10-F are much larger than those of PS10, which helps differentiate them. It can be concluded that the fluorochrome inside PS10-F contributes to the fluorescence, which leads to much larger F than that of PS10. This result indicates that the simultaneous measurement of polarized light scattering and fluorescence effectively enhances the discrimination ability of our method.

### 3.2. Different Categories of Microalgae

**Figure 4:**
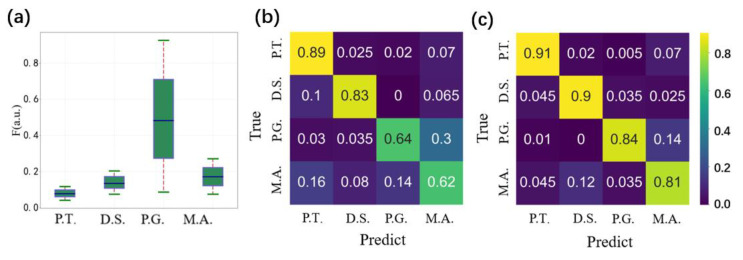
(**a**) Fluorescence distributions of different microalgae. (**b**) Classification confusion matrix using only polarization parameters. (**c**) Classification confusion matrix using both polarization and fluorescence parameters.

Four categories of microalgae were then compared and analyzed, namely P.T., D.S., P.G., and M.A.: their detailed information can be found in Section 2.1. Suspensions of these four microalgae were measured one by one in order to record the polarization and fluorescence parameters. Figure 4a shows the fluorescence distributions; the four categories of microalgae displayed different distributions of fluorescence. However, there are some overlaps among these categories, which results in difficulty in the classification of these four categories when the classification is only based on the fluorescence. The fluorescence intensity F of the light source was the highest for P.G. and the lowest for P.T., and the standard deviation of P.G. was also very large, which indicates a wide variation in the intracellular pigments.

The simultaneously recorded polarization parameters revealed the morphological and microstructural feature of these samples. To classify different categories of microalgae, we applied SVM to project the raw data to a higher dimension and realize the classification among these microalgae. Firstly, the input training data consisted of 1500 data points, and each data point comprised the five polarization parameters, [*I*, *q*, *u*, *v*, DOP]. The output result based on only polarization parameters is shown in the confusion matrix in Figure 4b. It can be seen that P.T. and D.S. were well classified using the polarization parameters, with accuracies of 83% and 89%, respectively. However, the classification accuracies of M.A. and P.G. are low, they are 64% and 62%, respectively. To take advantage of the fact that these four microalgae have different distributions of F, as shown in Figure 4a, we merged *F* with [*I*, *q*, *u*, *v*, DOP] to obtain six parameters, [*I*, *q*, *u*, *v*, DOP, *F*], in order to describe each cell. The resulting classification confusion matrix is shown in Figure 4c. Comparing Figure 4b,c, the classification accuracies of P.G. and M.A. were improved from 64% and 62% to 84% and 81%, respectively. Therefore, the combining of polarized light scattering and fluorescence effectively improved the microalgal classification.

### 3.3. Submicron Particles Measurement

**Figure 5:**
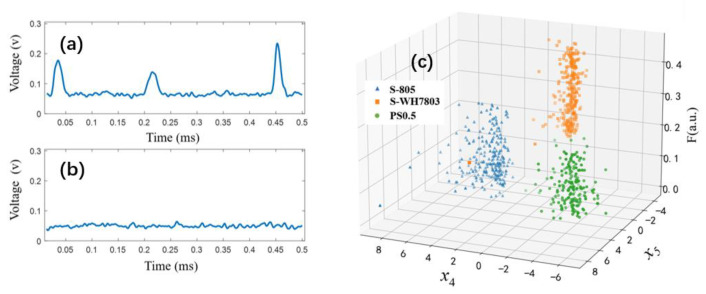
(**a**) Measured voltage signals of a PS microspheres of 0.5 μm (PS0.5). (**b**) Measured voltage signals of pure water (PW). (**c**) Distributions of three particle categories using both polarization parameters and fluorescence.

Submicron particles are extensively distributed in aquatic ecosystems. Note that the smallest observable size of microalgae in the ocean is approximately 0.5 μm. The setup was tested using an aquatic suspension of 0.5-μm-diameter PS microspheres (PS0.5). The temporal signals are shown in Figure 5a. Comparing these signals with that of pure water (PW) in Figure 5b, the signal-to-noise ratio of the pulses in Figure 5a originating from the scattering of PS0.5 is larger than 2, which implies that submicron suspended particles can be effectively detected by the setup.

*Synechococcus*, with a diameter of approximately one micron, is one of the smallest and most important cyanobacteria in seawater. However, the traditional method of identifying species of *Synechococcus* is analyzing their microscopic morphology and bulk fluorescence in the laboratory, and it is still challenging to in situ probe their individual cells in water. To check the ability of the setup to probe tiny particles, suspensions of PS0.5, and two species of *Synechococcus* 805 (S-805) and WH7803 (S-WH7803) were separately measured and analyzed. The distributions of these measured parameters are shown in Figure 5c, where x4 and x5 are two polarization parameters obtained using LDA. These three types of particles could be well classified using two polarization parameters and F value. This indicates that in situ probing of *Synechococcus* can be achieved by our method.

## 4. Discussions

The setup presented in this paper takes advantage of the high-power density of illumination light originating from the light focusing and the high sensitivity of the photoelectric detector in order to achieve simultaneous measurements of the scattered Stokes vector and the fluorescence of the individual microalgae. The relationship between the intracellular content of chlorophyll-a (Chl-a) and fluorescence intensity $F^{\prime }$ [$\mu \mathrm{mol}/\left(m^{3}\cdot s^{1}\right)$] is shown in Equation (5),

(5) $$F^{\prime }=PAR\cdot \left[chla\right]\cdot {\bar{a}}^{*}\cdot \;Q_{a}^{*}\left(\lambda_{em}\right)\cdot \phi_{F},$$

where *PAR* is the intensity of photosynthetically active radiation [$\mu \mathrm{mol}/\left(m^{2}\cdot s^{1}\right)$], [chla] is the Chl-a concentration ($mg\cdot m^{-3}$), ${\bar{a}}^{*}\;$is the absorption coefficient per chlorophyll concentration $\left(m^{2}\cdot mg^{-1}\right)$, $Q_{a}^{*}\left(\lambda_{em}\right)$ is the dimensionless fluorescence reabsorption factor, and $\phi_{F}$ is the emitted quantum efficiency factor.

For *Dunaliella salina* (D.S.) with the growth rate of 2.08 $d^{-1}$, PAR is 2.22 × 10^10^ $\mu \mathrm{mol}/\left(m^{2}\cdot s^{1}\right)$, and other related parameters in Equation (5) are given in the related report [27]. It can be calculated that the total fluorescence intensity is 9.75 nW. Using our data acquisition card, the corresponding received voltage is around 0.17 V, which is consistent with the experimental data. Furthermore, in future work, the chlorophyll content of a single cell may be determined by measuring the fluorescence of individual microalgae.

Laser irradiation may have a damaging effect on photosystem II and may inhibit the growth of algae when the exposure energy is larger than 816.43 μJ [28]. In our experimental setup, the light did not interact with algal cells for longer than 0.1 ms, and considering the energy density in the focal spot, the energy illuminating the algal cell was less than 0.1 μJ; therefore, there was no obvious destructive effect of laser irradiation on algal cells in the experiments.

The proposed prototype simultaneously measures the scattered polarization parameters and the fluorescence of the individual particles in water, which enables it to classify the particles into different categories and then determine their proportions. If we know the total concentration of the particles in water, then we can obtain the concentration of each particulate component. Different from the bulk measurements [11], the fluorescence of the individual particles, especially autofluorescence, is too low to measure, which is challenging for the community. In this work, we measured the overall fluorescence excited by the incident 445 nm light. Several kinds of intracellular pigments, including chlorophyll, carotenoids, and phycoerythrin, can absorb the 445 nm light, and all of them contribute to the detected fluorescence. If the spectrum of the fluorescence can be measured, except for the huge difficulty, the classification ability of this method may be advanced in the future.

## 5. Conclusions

In conclusion, this paper presented a portable prototype to simultaneously measure the polarized light scattering and fluorescence of individual particles. The experimental setup was introduced, and several experiments were conducted. The results demonstrated the ability of our method to in situ characterize various biological and non-biological particles, and demonstrated that classification can be realized. Moreover, it was shown that our setup can measure submicron particles and conduct classifications between different species of *Synechococcus*. The proposed technique is expected to be a powerful tool for marine ecological research and water quality monitoring.

## Data Availability

Data sharing not applicable.
